# The State’s Duty to the Orphan

**Published:** 1902-10

**Authors:** Edgar J. Spratling

**Affiliations:** Forsyth, Ga.


### THE STATE'S DUTY TO THE ORPHAN.*

By EDGAR J. SPRATLING, B.S., M.D.,
FORSYTH, GA.

When first asked to write a paper on this subject, a feeling of utter incompetency came over me. What presumption to discuss a theme of such depth and importance; to walk upon ground hardly explored by the oldest, wealthiest and most progressive governments. But acting on either, "the cat may look at the queen," or "fools rush in where angels fear to tread," I will try to arouse interest in this vital component of the State's future greatness.

What a field for harvests, broad as the horizon, fertile, fallow since times primeval, blessed by heaven's dews and sunshine. The richest field of charity open for the State's cultivation, let us pass in through its gate already ajar, and as we enter read on its arch, "As ye do unto the least of these, so do ye unto me," and find within the noblest task ever undertaken by the human hand and mind, the care and training of the helpless orphan.

Think how large a part of the future depends upon these little fellows, and whether that part be creditable or the reverse hangs on how we meet our obligations to them. They are the plastic clay, we the sculptors. Will noble, beautiful figures grow under our sympathetic touch, or ugly mud babies, because of our indifference? The strength of the State is the vigor of its people; its beauty is the beauty thereof, and it is ours to give it the greatest share of each. We men and women look back at our childhood and see the weaknesses of our training. Note where the clay of our natures was left rough, where the chiseling of our brains was hastily and badly finished, and then the impulse to improve upon the child beside us becomes irresistible. It is, we all know, the parent's duty to do this for his child. But oh, the pitiful little creature who has never said "father," and the far more to be pitied one who has never had the unspeakable boon of lisping

*Read before the Georgia Sociological Society, Atlanta, June, 1902

"mama." These belong to the State by every right, by every law divine, by every custom human.

When the Grand Monarch said to his ministers, "L'état, c'est moi," he expressed the status of every citizen of Georgia; then if we are the State, these little waifs are our children; their futures rest with us; it is ours to make them by lending the helping hand in this their time of greatest need, or mar them by resting idle, ignorant, careless and criminal as to their future. They ought to be, man for man, as important in the future greatness of Georgia as our own sons; they are to share, and share alike, with the sons of our bodies the trials and triumphs of our State.

Every nation, every State and every community is prosperous, happy and powerful as the average of its citizenry makes it. When we lead the boy from the squalid hut to the well-kept freeholder's cottage we have raised the plane of citizenship for that community. And when we take the foundling from the doorstep, making of him a leader among men; and the hungry babe from the cradle, forever stilled by the death palsy touching its mother's hand, making of her the companion of that leader of men, have we not used in its fullest sense that noblest attribute given by an all-wise providence to man alone? We have done unto others as we would have others do unto us. Surely man nor God could do more.

Now, how are we as a State to accomplish this great task? Are we to idly look on while individuals and religious denominations do our work in their own way? No, a thousand times no!

Far be it from me to decry the noble efforts of these unselfish men and women who are giving their lives' labors to this task. In the great hall of silent but eternal memory their names are writ in immutable figures, not graven on brass but etched on the skylights of eternity. No need have they for our praise. Happy immortality in the great world beyond renders all éclat among us needless and even out of place. They need not our praise, but, brothers, they suffer for our help. These consecrated men and women who are doing the State's duty, succoring the State's helpless little ones, are suffering by the lack of the State's aid. Yes, even of its encouragement. Not the grasp of a friendly hand do we the State extend to them. This is a shame upon us as an enlightened State; as a free people, it is a discredit to our nobler feelings, an injustice to the future; it is a crime towards our helpless wards.

The greatest objection to the present arrangement is the lack of authority to do for these wards what is really best for them. We know that some should be placed out by adoption, others by apprenticeship, many brighter ones in technical schools, a few in normal training institutes, and every one according to nature's light be given a chance for equality with others in the struggle of life. Only the State could afford such comprehensive paternalistic benefaction.

To many of the children asylum life as we find it to-day is a serious damage; it dwarfs individuality. The very uniform is a bar to personal aggressiveness. Discipline all but forbids independent initiative. They come to regard themselves as a mere mud clod on the wheel of progress instead of a spoke therein. They look at the children on the outside as being apart from themselves, having nothing in common, not a heart throb in unison. And the outside children regard them somewhat as they would monkeys in a cage. That is a rough picture, but it has the true coloring.

The orphan and the foundling, to become the peer of his more fortunate brother, must be raised in like or superior manner, to him; if possible brought up in home, at school, in workshop, as an individual and not as part of a living automaton. Give him advantages that will obliterate the stigma of having been charity reared, and that will avoid the curse of having been turned loose on society a pauper.

By a pauper I do not mean one without money, but one without the capacity of earning a support. I have yet to know the orphan or foundling purely asylum raised, under the conditions pertaining in Georgia, who has risen even to comparative prominence. On the other hand, we all know successful ones who lived in private homes.

If dying to-day I were leaving a son to be raised in an average orphan asylum, my last breath would be a prayer to the God of kindness that that child go to the grave with me.

Let me speak a moment of the experience of New York City. Till 1899, the foundlings were taken to the City Asylum on the Island. In 1897, the mortality compared to admissions was 98 per cent. In 1898, it was 98.6 per cent. In 1899, it actually closed house with 100 per cent. of deaths. What a record. No wonder New York City felt a change demanded. And what change? Simply placing out in families. Result? A mortality the first year, 1900, of 55 per cent. Think of it, 45 per cent. of these little lives saved that year. And the next? Why, the mortality fell to 33 per cent. Isn't that grand. Furthermore, the city saved money; for the cost of maintenance in families was less than it would have been in the Asylum.

When we remember that the majority of these foundlings are natural children, who are brighter mentally and fitter physically than legitimate children of like parental status, and far, very far, superior to the average orphan, we realize what an enormous impetus the city has gained for future progress, in the saving of these little creatures.

A child suitably placed in a family becomes an integral part of it. It shares the joys, hopes, successes and failures. It learns life as life really is. It sees the world in vision clear and not as a smoky panorama; feels the impulses of normal life; feels and knows itself a part of the great forward moving drama about it, not standing on a pedestal separated by that impassable barrier, asylum rules and asylum uniform, from the history-making tide of human affairs.

As a nucleus the State should use the asylums already established thus: Assign to each a certain district, making the commitment to them direct from the ordinaries' courts, and the child's reception at the asylum mandatory, the State paying a fair rate per capita for maintenance. When enough of these State charges have been accumulated in the various asylums to warrant it, let them all be collected in one institution, combining the features of asylum, school, manual training-school, and even technological institute. Yes, and further if demanded, reformatory. Its charter should provide for the placing out of children by adoption, on probation, in apprenticeship, and sending away for special training or the development of particular talents. My! how I'd love to be a trustee of such an institution. The above applies to the normal and superior children; for defective ones there must be special arrangements.

Every child wherever located should be seen at least once each quarter by a State Inspector, whose sole duty and care should be the intelligent observation of each one individually, and reporting minutely to a central board of public charities.

As a money investment this is the best that could be made—every able-bodied working man is worth to society $400.00 a year. Every working woman is worth $200.00. Those specially skilled are worth corresponding amounts. Now, we have the opportunity of making not only laborers, but skilled artisans of a large proportion of the orphans and foundlings of Georgia; and we all know that the most urgent need of this fair Southland is skilled labor. Will we let slip by this chance of making social ornaments of those who are now economic parasites?

Even the defectives can ofttimes be made more than self-supporting. They should be taught handicrafts, as well as farming, gardening, brickmaking and other things useful. It is wonderful how competent an imbecile of even the second or third degree can be made by careful training. Even an idiot of low degree may be made self-supporting by being taught rough labor. Those most abject pieces of humanity, those poor creatures avoided by men and seemingly neglected of God, the epileptics, can, as has been proven at Craig Colony, New York, and Bielefeld, Westphalia, be very nearly independent industrial factors.

Think of what an incorruptible treasure in loyalty and patriotism the State will lay up in these men and women fostered, nurtured, reared, educated and trained by it! Why the State will persist in lavishing its bounty and exhausting its charitable skill on the insane and ignore the Macedonian cry of the orphan passes my understanding. Why spend all in propping up the crumbling turret while the very foundation sinks in sheer neglect? The addition to the already heavy negro burden, in assuming care of the orphans, is the one real drawback. But when we remember how cheaply they can be cared for, either in asylum or boarded out, that objection becomes much less. Then, too, humanity must ever play a part in dealing with this subject.

Let this association, individually and collectively, wake up to this noblest and most needed of public charities, that we may help to guide the State's footsteps into this path leading so certainly, so directly, to an increase of the majesty and glory of our beloved Georgia.

